# Supplementary material for: Self-propelled assembly of nanoparticles with self-catalytic regulation for tumour-specific imaging and therapy
Source: Nat Commun. 2024 Jan 11;15:460. doi: 10.1038/s41467-024-44736-y (PMC10784296; doi:10.1038/s41467-024-44736-y)
Supplement: Supplementary file 1 — Supplementary Information [file 41467_2024_44736_MOESM1_ESM.pdf]

Supplementary Information

Self-Propelled Assembly of Nanoparticles with Self-Catalytic Regulation for Tumour-Specific Imaging and Therapy

Mengmeng Xia[1,8], Qiyue Wang[2,8], Yamin Liu[2,8], Chunyan Fang[2,8], Bo Zhang[2,7], Shengfei Yang[3], Fu Zhou[1], Peihua Lin[2], Mingzheng Gu[1], Canyu Huang[2], Xiaojun Zhang[1], Fangyuan Li[3,4,5,*], Hongying Liu[6,*], Guangfeng Wang[1,*], Daishun Ling[2,7,*]

[1]School of Chemistry and Materials Science, Anhui Province Key Laboratory of Biomedical Materials and Chemical Measurement, Center for Nano Science and Technology, Anhui Normal University, Wuhu 241000, China

[2]Frontiers Science Center for Transformative Molecules, School of Chemistry and Chemical Engineering, School of Biomedical Engineering, National Center for Translational Medicine, State Key Laboratory of Oncogenes and Related Genes, Shanghai Jiao Tong University, Shanghai 200240, China

[3]Institute of Pharmaceutics, Hangzhou Institute of Innovative Medicine, College of Pharmaceutical Sciences, Zhejiang University, Hangzhou 310058, China

[4]Key Laboratory of Precision Diagnosis and Treatment for Hepatobiliary and Pancreatic Tumor of Zhejiang Province, Hangzhou 310009, China

[5]Songjiang Institute and Songjiang Hospital, Shanghai Jiao Tong University School of Medicine, Shanghai, China

[6]College of Automation, Hangzhou Dianzi University, Hangzhou, 310018, China

[7]World Laureates Association (WLA) Laboratories, Shanghai 201203, China

[8] These authors contributed equally: Mengmeng Xia, Qiyue Wang, Yamin Liu, Chunyan Fang

Correspondence and requests for materials should be addressed to D.L., G.W., H.L., and F.L.

(*e-mail: dsling@sjtu.edu.cn; wangyuz@mail.ahnu.edu.cn; liuhongying@hdu.edu.cn; lfy@zju.edu.cn)

Contents

Supplementary Fig.1: TEM images of monodisperse CuS-I NPs and CuS NPs··················2
Supplementary Fig.2: The energy dispersive X-ray spectrum of CuS-I NPs··················2
Supplementary Fig.3: HRTEM image of CuS NPs··················2
Supplementary Fig.4: EPR spectra of CuS NPs and CuS-I NPs··················2
Supplementary Fig.5: XPS of CuS NPs and CuS-I NPs··················3
Supplementary Fig.6: FT-IR spectra and UV-Vis absorbance spectra of CuS-I NPs, P1 and CuS-I@P1 NPs··················3
Supplementary Fig.7: DLS of CuS-I@P1, CuS-I@P1-scr and CuS@P1··················3
Supplementary Fig.8: Schematic illustration of intracellular routes of CuS@P1 and CuS-I@P1-scr NPs and corresponding FT-IR spectrum··················4
Supplementary Fig.9: The TEM images of different CuS-I@Tyr-X··················4
Supplementary Fig.10: TEM images of CuS-I@P1 incubated with furin-H_2O_2 for different time··················5
Supplementary Fig.11: FRET between CuS-I NPs and FAM··················5
Supplementary Fig.12: Fluorescence spectrum of FAM after CuS-I@P1-scr NPs incubated with furin····5
Supplementary Fig.13: The standard curve of P1··················5
Supplementary Fig.14: UV-Vis absorbance and photothermal conversion effect of nanoparticles··················6
Supplementary Fig.15: The absorbance of CuS-I NPs and CuS-I NAs··················6
Supplementary Fig.16: The dependence of $(Ahv)^{1/2}$ on hv for CuS-I NPs and CuS-I NAs··················7
Supplementary Fig.17: CLSM images and the Pearson's correlation coefficient of the MDA-MB-468 cells incubated with CuS-I@P1 NPs··················7
Supplementary Fig.18: TEM image and fluorescence intensity MDA-MB-468 cell lysate··················7
Supplementary Fig.19: Bio-TEM images of MDA-MB-468 cells with different treatments··················8
Supplementary Fig.20: Confocal fluorescence images of 293T cells··················8
Supplementary Fig.21: Time-dependent infrared thermal images and temperature changes at the tumour site administered with CuS-I@P1··················9
Supplementary Fig.22: Time-dependent infrared thermal images and temperature changes at the tumour site administered with CuS@P1··················9
Supplementary Fig.23: Time-dependent infrared thermal images and temperature changes at the tumour site administered with CuS-I@P1-scr··················9
Supplementary Fig.24: Bio-TEM images of tumour sections from MDA-MB-468-tumour-bearing mice post administration of CuS-I@P1 NPs··················10
Supplementary Fig.25: Bio-TEM images of tumour sections from MDA-MB-468-tumour-bearing mice post administration of different groups··················10
Supplementary Fig.26: H&E staining images of major organs··················11
Supplementary Fig.27: Hematology analysis of mice from different groups··················12
Supplementary Table 1: ··················12

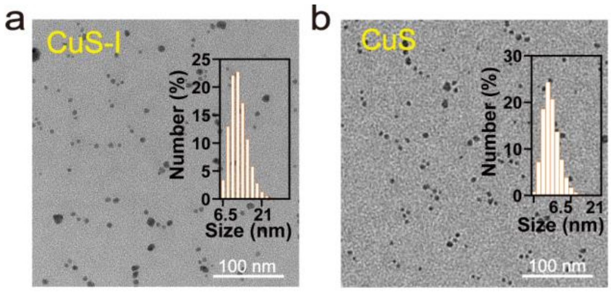

Supplementary Fig.1. Transmission electron microscope (TEM) images of monodisperse CuS-I NPs (a) and CuS NPs (b).

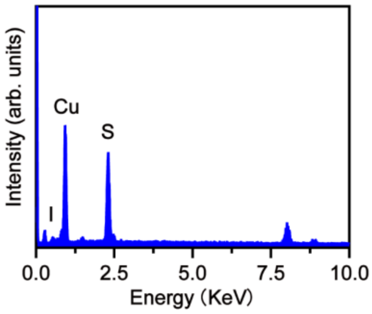

Supplementary Fig.2. The energy dispersive X-ray spectrum of CuS-I NPs.

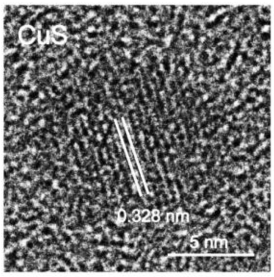

Supplementary Fig.3. High resolution TEM image of CuS NPs.

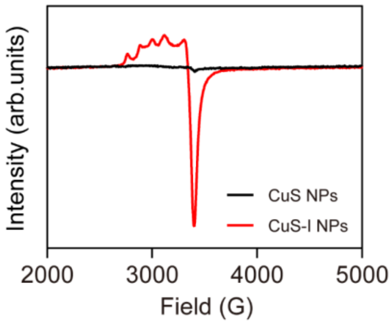

Supplementary Fig. 4. Electron paramagnetic resonance (EPR) spectra of CuS NPs and CuS-I NPs.

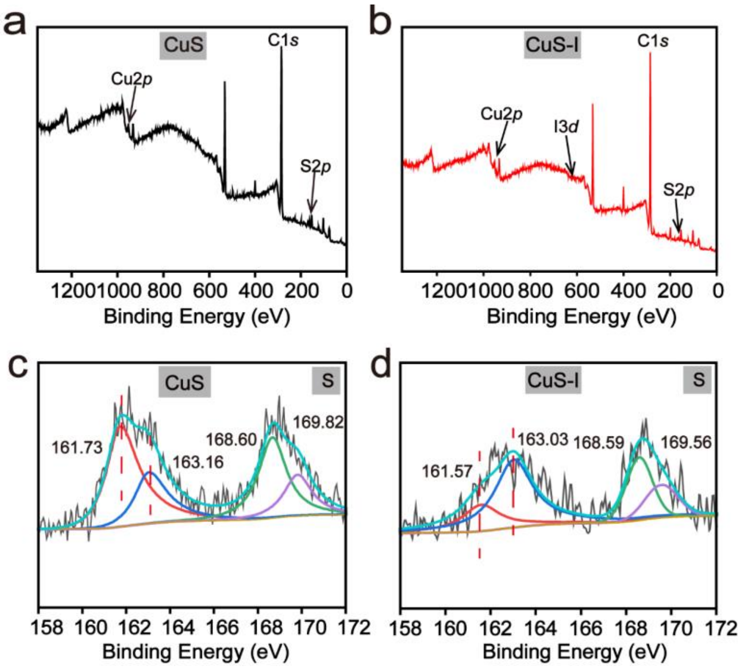

Supplementary Fig.5. X-ray photoelectron spectroscopy (XPS) spectra of CuS NPs (a) and CuS-I NPs (b). High-resolution XPS spectra of S 2p in the CuS NPs (c) and CuS-I NPs (d).

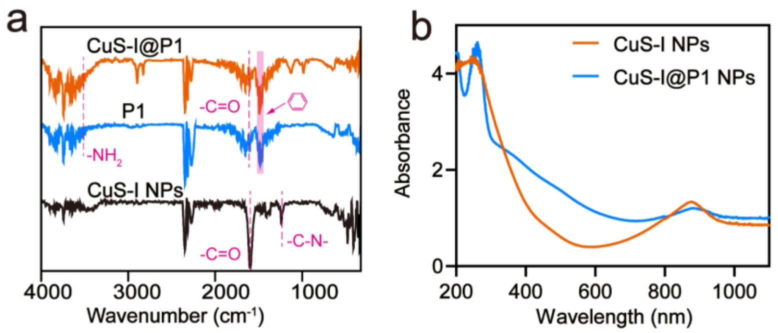

Supplementary Fig.6. (a) Fourier infrared (FT-IR) spectrum of CuS-I NPs, P1 and CuS-I@P1 NPs. (b) Ultraviolet visible (UV-Vis) absorbance spectrum of CuS-I NPs and CuS-I@P1 NPs.

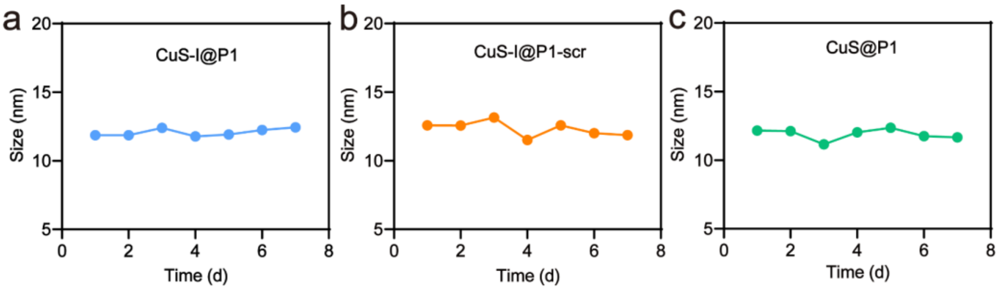

Supplementary Fig.7. Dynamic light scattering (DLS) of CuS-I@P1 (a), CuS-I@P1-scr (b) and CuS@P1 (c) in dulbecco's modified eagle medium containing 10% fetal bovine serum within 7 days ([CuS-I@P1] = [CuS-I@P1-scr] = [CuS@P1] = 200 μg mL^{-1}).

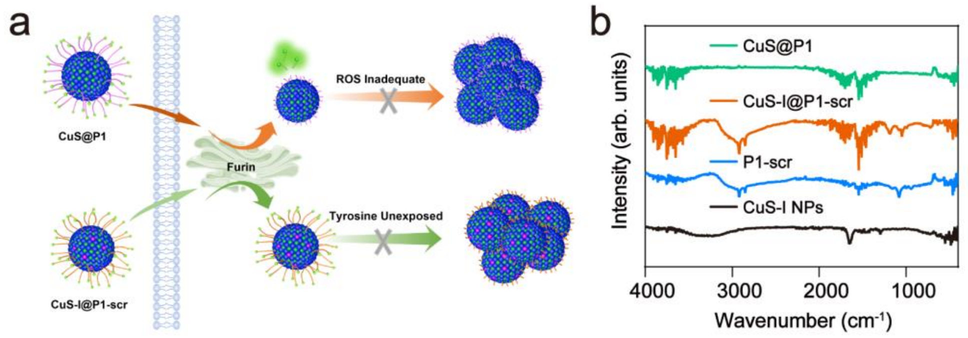

Supplementary Fig.8. (a) Schematic illustration of intracellular routes of CuS@P1 and CuS-I@P1-scr NPs. (b) FT-IR spectrum of CuS-I NPs, P1-scr, CuS@P1 and CuS-I@P1-scr NPs.

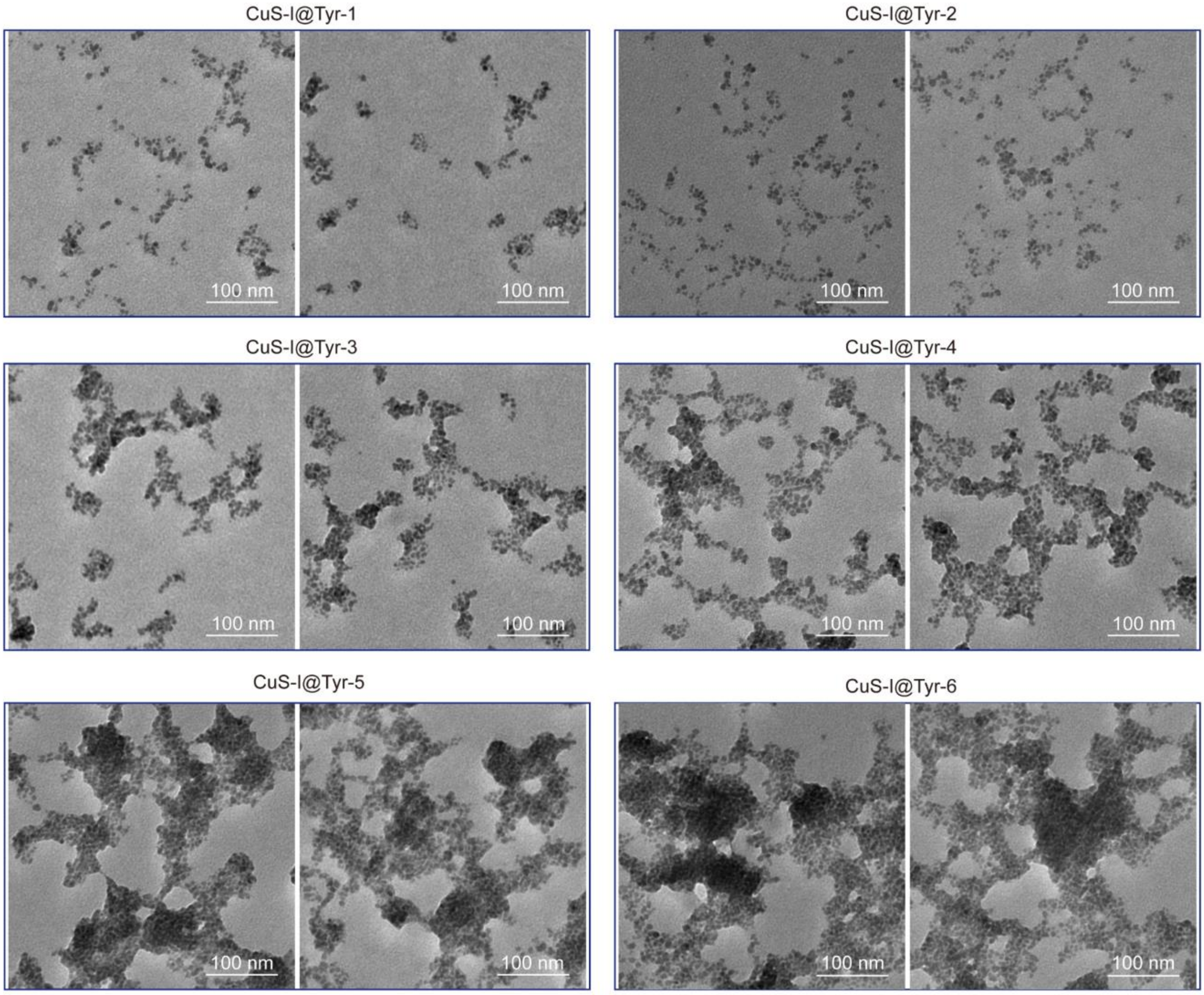

Supplementary Fig.9. TEM images of CuS-I NPs with distinct Tyr contents upon treatment with H_2O_2, which were prepared by adding varying volumes of a 0.55 mM Tyr solution (10 μL, 20 μL, 40 μL, 60 μL, 80 μL, 100 μL), denoted as CuS-I@Tyr-1, CuS-I@Tyr-2, CuS-I@Tyr-3, CuS-I@Tyr-4, CuS-I@Tyr-5, and CuS-I@Tyr-6, respectively.

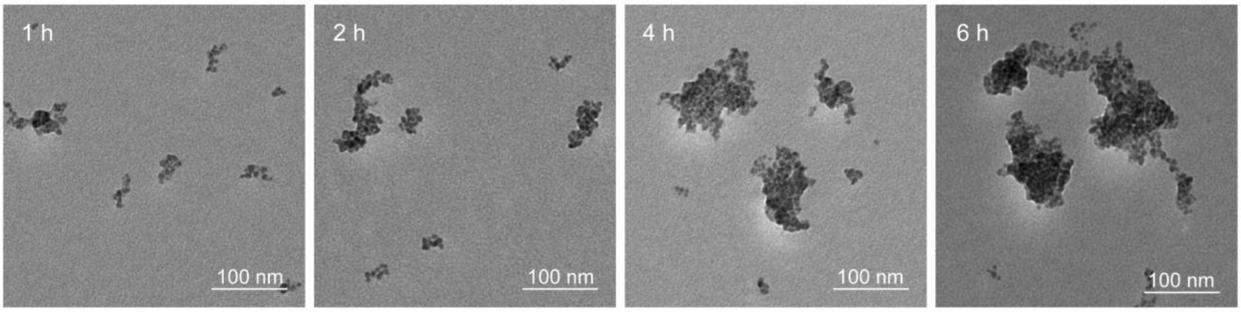

Supplementary Fig.10. TEM images of CuS-I@P1 incubated with furin and H_2O_2 for different times ([CuS-I@P1] = 200 μg mL^{-1}, [H_2O_2] = 100 μM, [furin] = 20 U mL^{-1}).

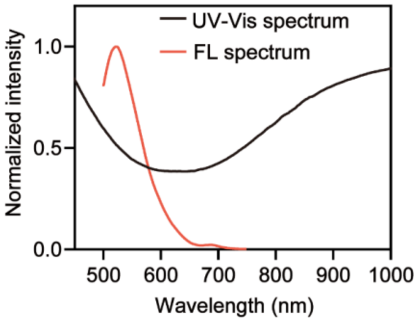

Supplementary Fig.11. UV-Vis absorption spectrum of CuS-I NPs and fluorescence emission spectrum of FAM.

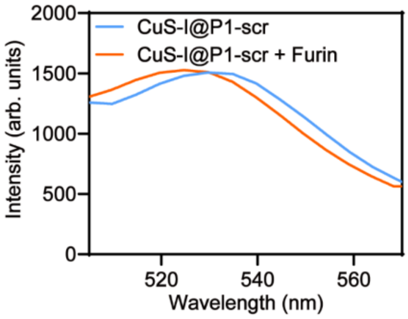

Supplementary Fig.12. Fluorescence spectrum of FAM after CuS-I@P1-scr NPs incubated with furin in the HEPES buffer ([CuS-I@P1-scr] = 200 μg mL^{-1}, [furin] = 20 U mL^{-1}).

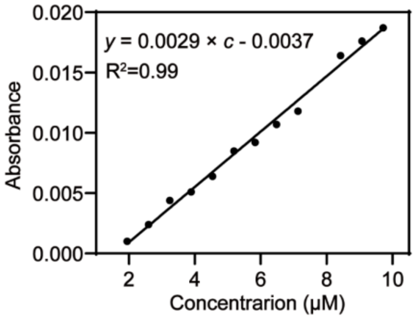

Supplementary Fig.13. The standard curve of P1.

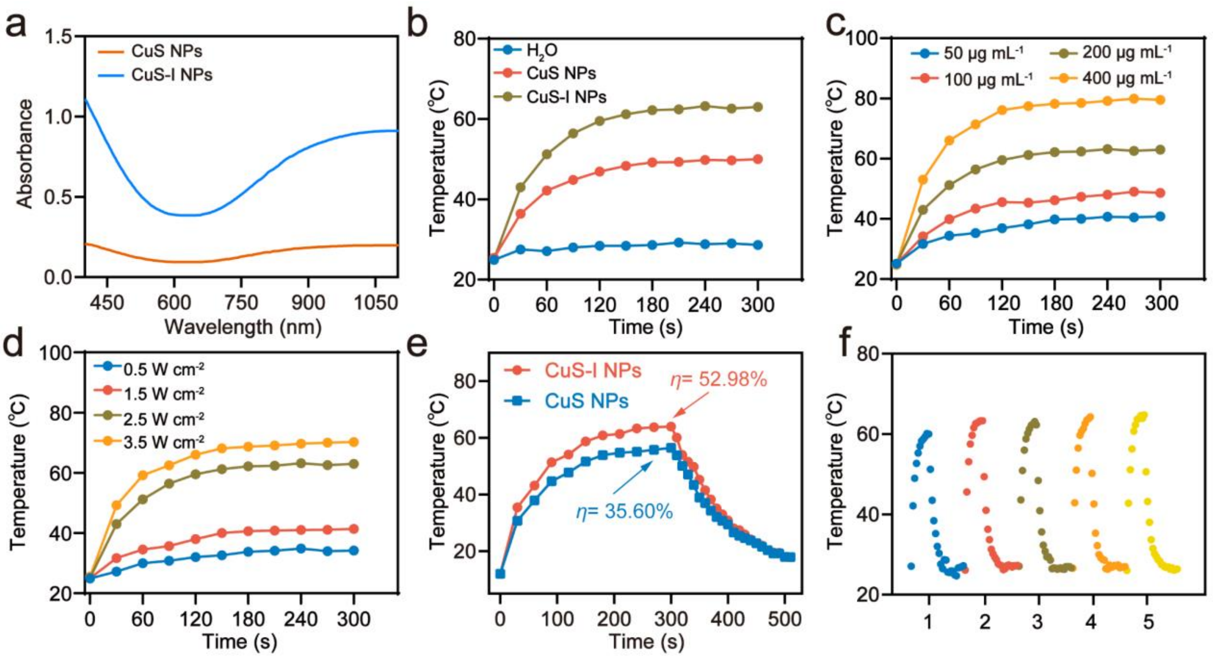

Supplementary Fig.14. (a) UV-Vis absorbance spectra of CuS NPs and CuS-I NPs. ([CuS] = [CuS-I] = 200 μg mL^{-1}) (b) Temperature change curves of CuS and CuS-I solution upon the laser irradiation (([CuS] = [CuS-I] = 200 μg mL^{-1}, 808 nm, 2 W cm^{-2}, 5 min). (c) Temperature change curves of CuS-I solution at various concentrations. (d) Temperature change curves of CuS-I solution at different power densities ([CuS-I] = 200 μg mL^{-1}). (e) Temperature changes of CuS-I and CuS solution under 300 s irradiation and subsequent 200 s cooling ([CuS] = [CuS-I] = 200 μg mL^{-1}). (f) Temperature change curves of CuS-I solution over five laser irradiation on/off cycles ([CuS-I] = 200 μg mL^{-1}, 808 nm, 2 W cm^{-2}).

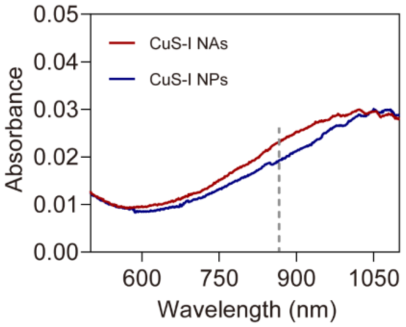

Supplementary Fig.15. The absorption spectra of CuS-I NPs and CuS-I NAs.

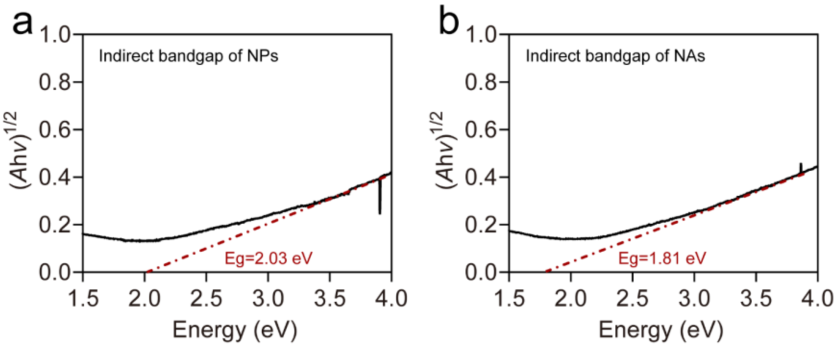

Supplementary Fig.16. Band gaps (Eg) for CuS-I NPs and CuS-I NAs were determined using the Tauc plot method.

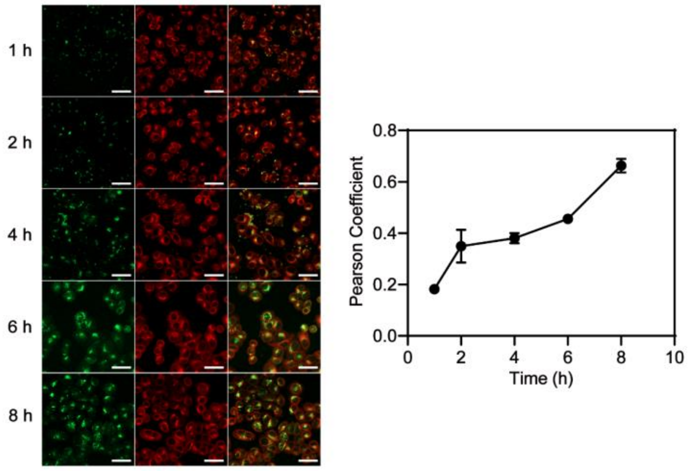

Supplementary Fig.17. (a) Confocal laser scanning microscopy (CLSM) images of the MDA-MB-468 cells incubated with CuS-I@P1 NPs (200 μg mL^{-1}, 1, 2, 4, 6, 8 h). Scale bar: 50 μm. (b) Pearson's correlation coefficient between CuS-I@P1 and Golgi bodies when the MDA-MB-468 cells incubated with CuS-I@P1 NPs at different time intervals (n = 5).

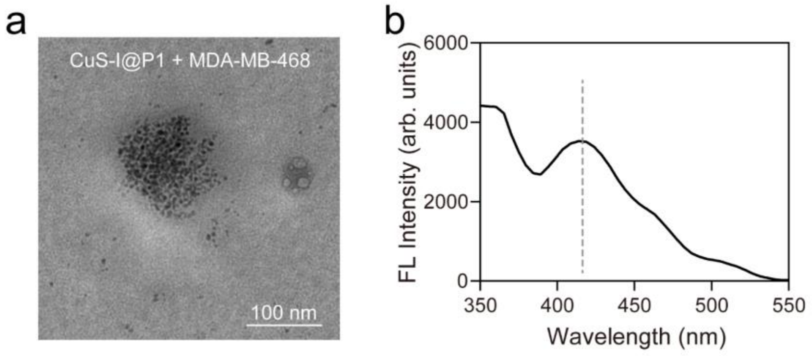

Supplementary Fig.18. (a) TEM image of the MDA-MB-468 cell lysate after incubation with CuS-I@P1 NPs for 8 h at 37 °C; (b) The fluorescence spectrum of the lysate of MDA-MB-468 cells, following an 8-hour incubation with CuS-I@P1 NPs at 37 °C, exhibits a characteristic fluorescence signal of dityrosine at ~ 410 nm.

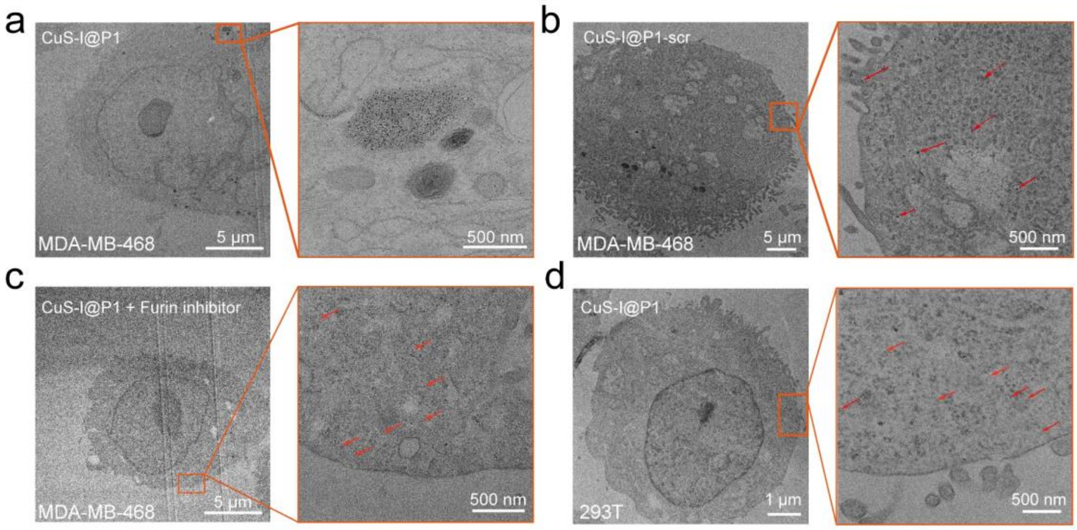

Supplementary Fig.19. Low magnification (left) and high magnification (right) bio-TEM images of MDA-MB-468 cells incubated with CuS-I@P1 NPs (a), CuS-I@P1-scr NPs (b), furin inhibitor II (10 mM) for 1 h and then CuS-I@P1 NPs (c) for 8 hours at 37 °C. Red arrows in (b) and (c) indicate CuS-I@P1-scr NPs and CuS-I@P1 NPs, respectively. ([CuS-I@P1] = [CuS-I@P1-scr] = 200 μg mL^{-1}); (d) Low magnification (left) and high magnification (right) bio-TEM images of 293T cells after incubation with CuS-I@P1 NPs for 8 h at 37 °C. Red arrows point to the CuS-I@P1 NPs. ([CuS-I@P1] = 200 μg mL^{-1}).

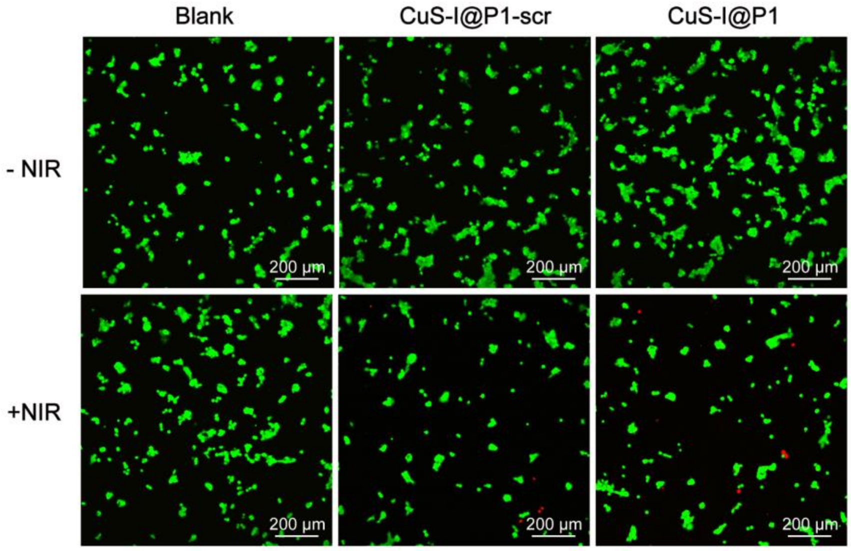

Supplementary Fig.20. Confocal fluorescence images of 293T cells co-stained with calcein AM (green, live cells) and propidium iodide (red, dead cells) after treated with phosphate buffer solution (PBS), CuS-I@P1, CuS-I@P1-scr with or without NIR laser irradiation ([CuS-I@P1] = [CuS-I@P1-scr] = 200 μg mL^{-1}).

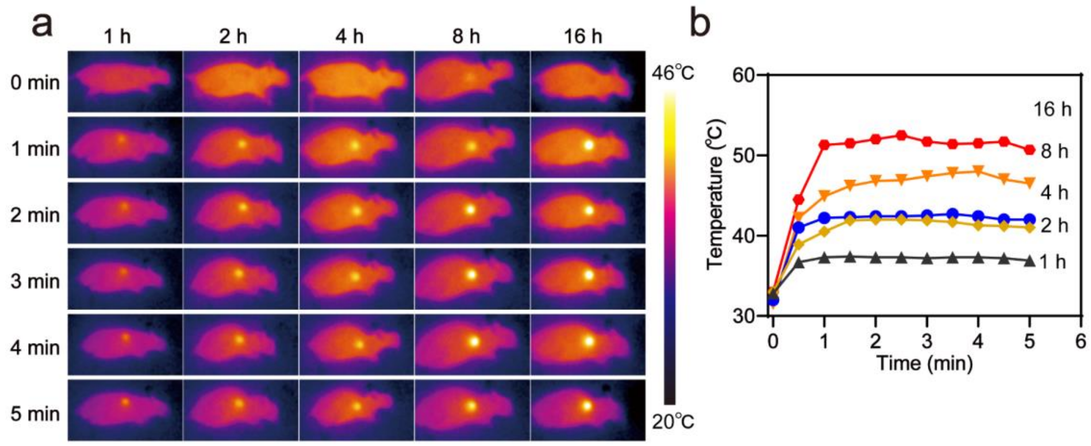

Supplementary Fig.21. Time-dependent infrared thermal images (a) and temperature changes at the tumour site (b) of MDA-MB-468 tumour-bearing mouse subjected to laser irradiation (808 nm, 2 W cm^{-2}, 5 min) at 1 h, 2 h, 4 h, 8 h, or 16 h after being intratumourally administered with CuS-I@P1 (3 mg mL^{-1}, 50 μL) dispersed in PBS.

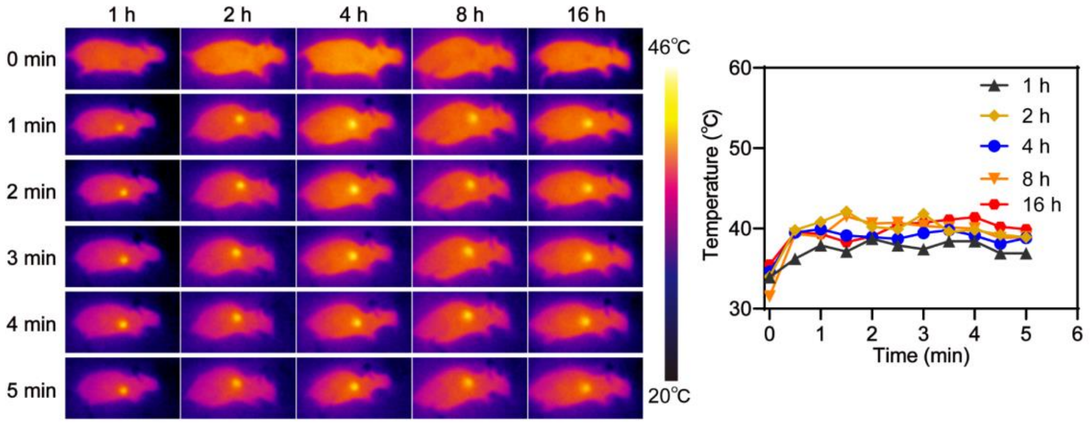

Supplementary Fig.22. Time-dependent infrared thermal images of MDA-MB-468 tumour-bearing mouse subjected to laser irradiation (808 nm, 2 W cm^{-2}, 5 min) at 1 h, 2 h, 4 h, 8 h, or 16 h after being intratumourally administered with CuS@P1 (3 mg mL^{-1}, 50 μL) dispersed in PBS.

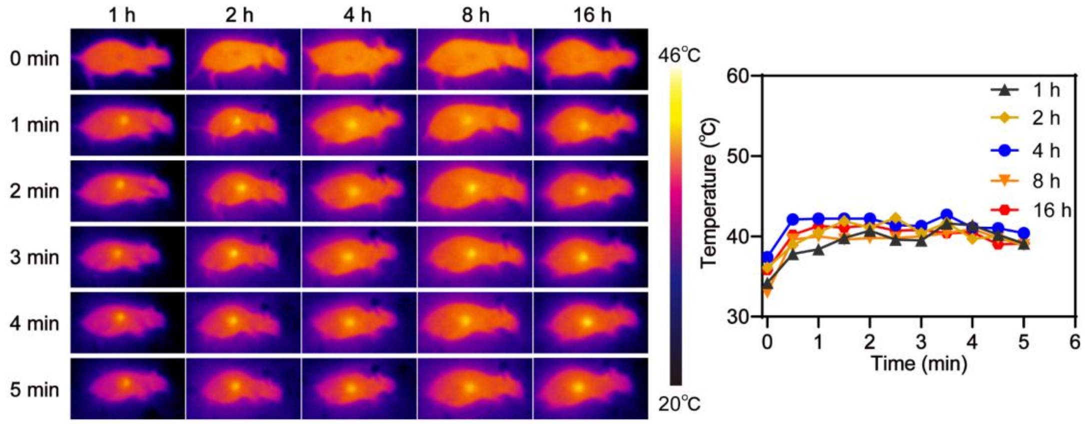

Supplementary Fig.23. Time-dependent infrared thermal images of MDA-MB-468 tumour-bearing mouse subjected to laser irradiation (808 nm, 2 W cm^{-2}, 5 min) at 1 h, 2 h, 4 h, 8 h, or 16 h after being intratumourally administered with CuS-I@P1-scr (3 mg mL^{-1}, 50 μL) dispersed in PBS.

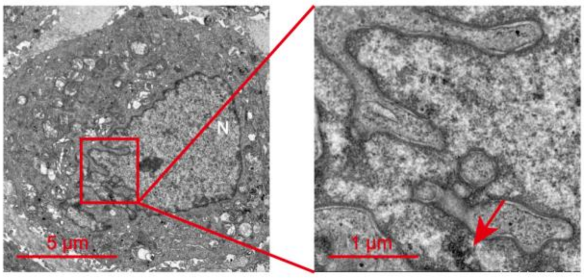

Supplementary Fig.24. Bio-TEM images of tumour sections from MDA-MB-468-tumour-bearing mice at 8 h post intratumoural administration of CuS-I@P1 NPs dispersed in PBS ([CuS-I@P1] = 3 mg mL^{-1}). The right is the high magnification bio-TEM image of the red rectangle area in a. Red arrow point to the CuS-I NAs.

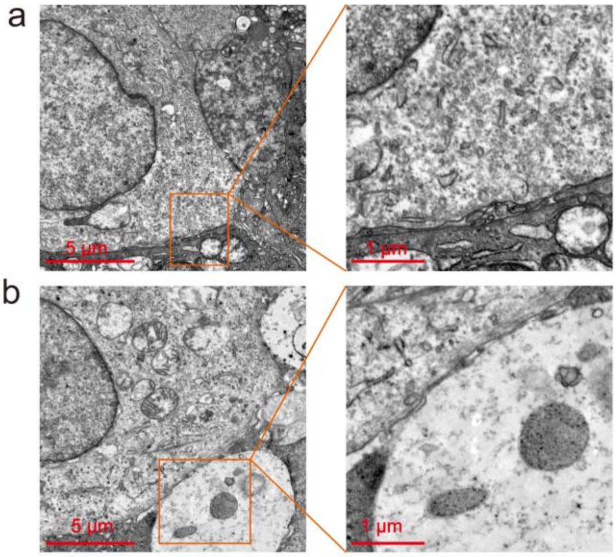

Supplementary Fig.25. Bio-TEM images of tumour sections from MDA-MB-468 tumour-bearing mice at 8 h post intratumoural administration of CuS-I@P1-scr NPs (a), or CuS@P1 NPs (b) dispersed in PBS ([CuS@P1] = [CuS-I@P1-scr] = 3 mg mL^{-1}). The right image in a/b is the high magnification bio-TEM image of the orange rectangle area in the left image in a/b, respectively.

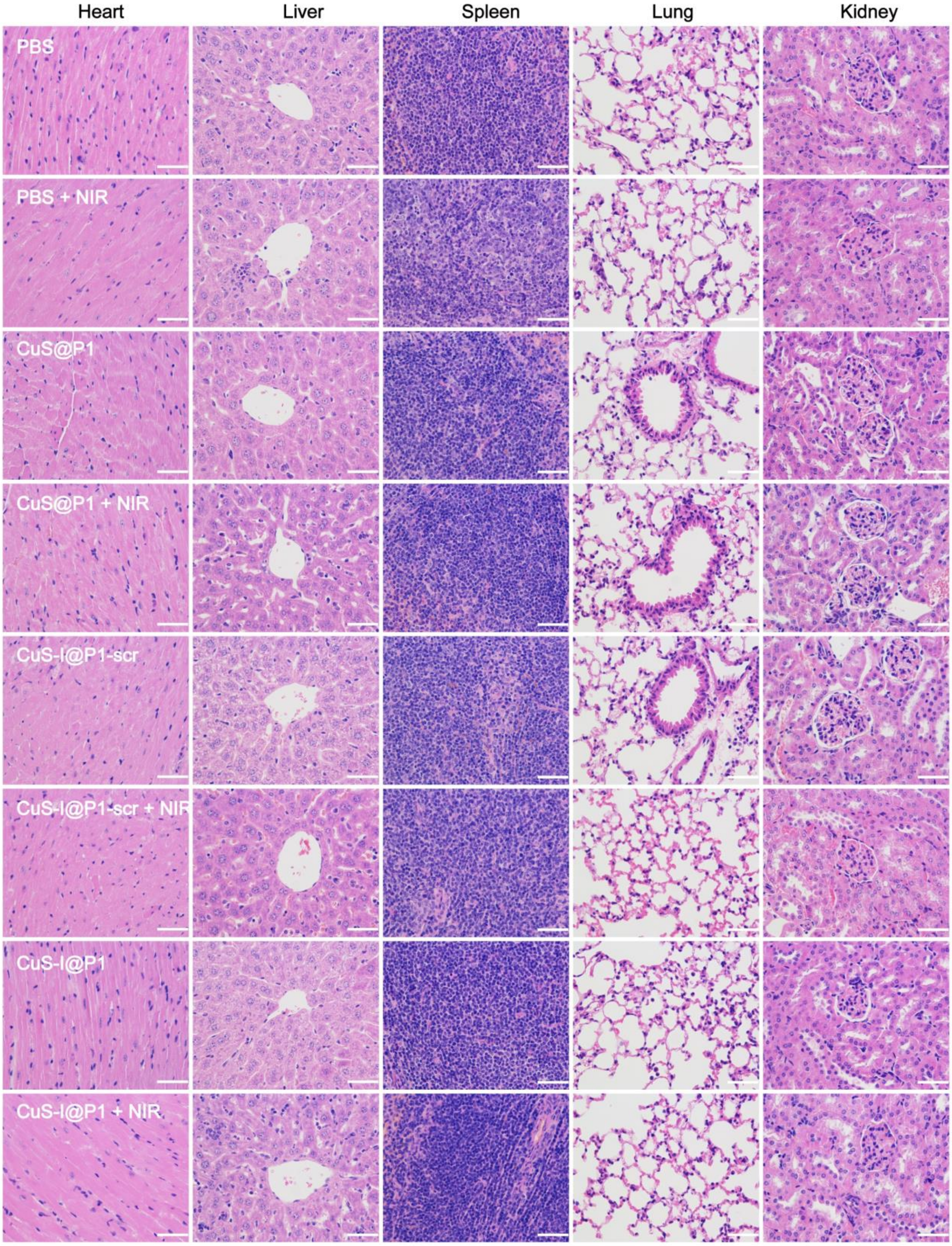

Supplementary Fig.26. H&E staining images of major organs. Scale bar: 50 μm.

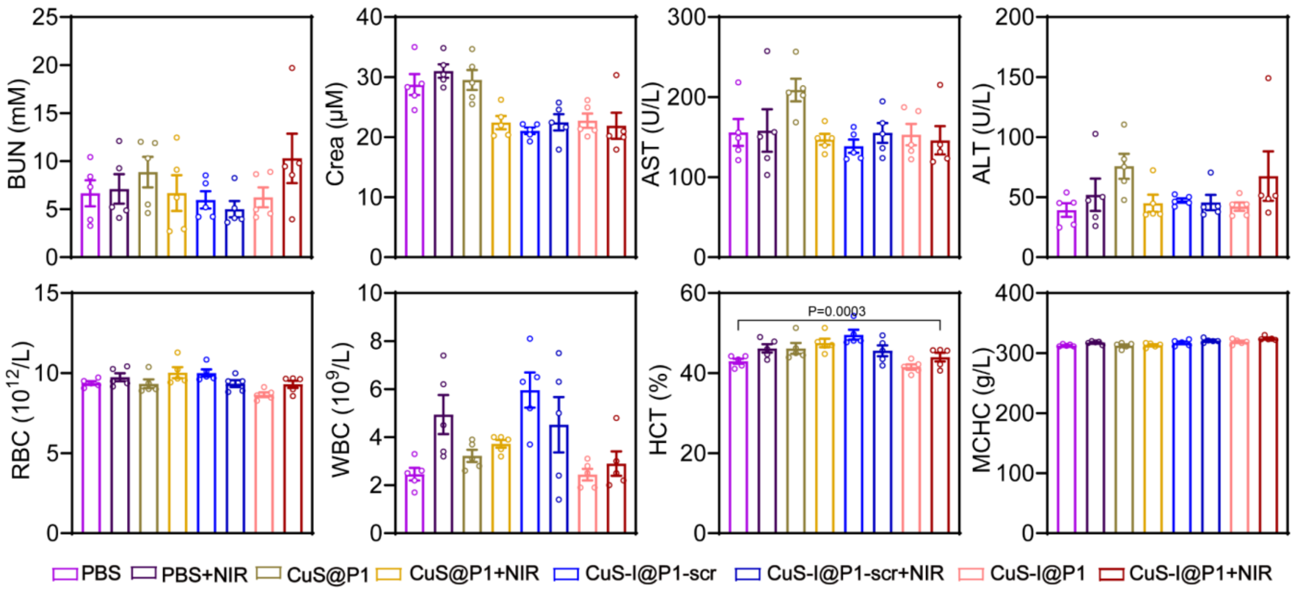

Supplementary Fig.27. Hematology analysis of mice from different groups at day 36 post-injection (BUN: blood urea nitrogen; Crea: creatinine; AST: aspartate aminotransferase; ALT: alanine aminotransferase; RBC: red blood cell; WBC: white blood cell; HCT: hematocrit; MCHC: mean corpuscular hemoglobin concentration). (*n* = 5).

Supplementary Table 1

Samples	Molar concentration of S (mg mL^{-1})	Molar concentration of I (mg mL^{-1})	I/(I+S) ratio
CuS-I	104.2	1.2	1.14%
