# Supplementary material for: Self-propelled assembly of nanoparticles with self-catalytic regulation for tumour-specific imaging and therapy
Source: Nat Commun. 2024 Jan 11;15:460. doi: 10.1038/s41467-024-44736-y (PMC10784296; doi:10.1038/s41467-024-44736-y)
Supplement: Supplementary file 2 — Reporting Summary [file 41467_2024_44736_MOESM2_ESM.pdf]

nature portfolio

Corresponding author(s): Daishun Ling

Last updated by author(s): Dec 20, 2023

Reporting Summary

Nature Portfolio wishes to improve the reproducibility of the work that we publish. This form provides structure for consistency and transparency in reporting. For further information on Nature Portfolio policies, see our Editorial Policies and the Editorial Policy Checklist.

Statistics

For all statistical analyses, confirm that the following items are present in the figure legend, table legend, main text, or Methods section.

n/a	Confirmed	
[ ]	[x]	The exact sample size (*n*) for each experimental group/condition, given as a discrete number and unit of measurement
[ ]	[x]	A statement on whether measurements were taken from distinct samples or whether the same sample was measured repeatedly
[ ]	[x]	The statistical test(s) used AND whether they are one- or two-sided<br>*Only common tests should be described solely by name; describe more complex techniques in the Methods section.*
[x]	[ ]	A description of all covariates tested
[x]	[ ]	A description of any assumptions or corrections, such as tests of normality and adjustment for multiple comparisons
[ ]	[x]	A full description of the statistical parameters including central tendency (e.g. means) or other basic estimates (e.g. regression coefficient) AND variation (e.g. standard deviation) or associated estimates of uncertainty (e.g. confidence intervals)
[ ]	[x]	For null hypothesis testing, the test statistic (e.g. *F*, *t*, *r*) with confidence intervals, effect sizes, degrees of freedom and *P* value noted<br>*Give P values as exact values whenever suitable.*
[x]	[ ]	For Bayesian analysis, information on the choice of priors and Markov chain Monte Carlo settings
[x]	[ ]	For hierarchical and complex designs, identification of the appropriate level for tests and full reporting of outcomes
[ ]	[x]	Estimates of effect sizes (e.g. Cohen's *d*, Pearson's *r*), indicating how they were calculated

Our web collection on statistics for biologists contains articles on many of the points above.

Software and code

Policy information about availability of computer code

Data collection	Characterization of nanomaterials properties were carried out from transmission electron microscopy (Hitachi HT7700, Japan), energydispersive spectroscopy (Hitachi S-4800), Ultraviolet-visible absorption (Hitachi U-4100, Japan), Fluorescence spectrophotometer (Thermo scientific), Fourier transform infrared (Shimadzu FTIR-84005, Japan), XRD (SmartLab 9KWSmartLab 9KW, Japan), X-ray photoelectron spectrometer (Escalab 250Xi, Thermo Fisher), DLS and Zeta potential (Malvern Zeta sizer Nano-ZS ZEM3600, U.K.), high-resolution transmission electron microscopy (Thermofisher, USA),The laser used in this study (808 nm, Leirui Optoelectronics Co., Ltd., China), Infrared thermal imager (Fluke TiS10), Confocal images were captured by confocal laser scanning microscopy (Zeiss, Germany). High-performance liquid chromatography analyses were recorded on the Shimadzu chromatographic system (Tokyo, Japan).
Data analysis	Quantitative analysis of the fluorescence images was performed using Image J. Statistical comparisons were analyzed using OriginPro Learning Edition and Graphpad Prism (Version 8.0). The density functional theory calculations were carried out using Quantum Espresso (Version 6.7).

For manuscripts utilizing custom algorithms or software that are central to the research but not yet described in published literature, software must be made available to editors and reviewers. We strongly encourage code deposition in a community repository (e.g. GitHub). See the Nature Portfolio guidelines for submitting code & software for further information.

Data

Policy information about availability of data

All manuscripts must include a data availability statement. This statement should provide the following information, where applicable:

- Accession codes, unique identifiers, or web links for publicly available datasets
- A description of any restrictions on data availability
- For clinical datasets or third party data, please ensure that the statement adheres to our policy

The data generated in this study are provided in the Source Data file. All other data are available from the corresponding authors on request.

Research involving human participants, their data, or biological material

Policy information about studies with human participants or human data. See also policy information about sex, gender (identity/presentation), and sexual orientation and race, ethnicity and racism.

Reporting on sex and gender	N/A
Reporting on race, ethnicity, or other socially relevant groupings	N/A
Population characteristics	N/A
Recruitment	N/A
Ethics oversight	N/A

Note that full information on the approval of the study protocol must also be provided in the manuscript.

Field-specific reporting

Please select the one below that is the best fit for your research. If you are not sure, read the appropriate sections before making your selection.

[x] Life sciences [ ] Behavioural & social sciences [ ] Ecological, evolutionary & environmental sciences

For a reference copy of the document with all sections, see nature.com/documents/nr-reporting-summary-flat.pdf

Life sciences study design

All studies must disclose on these points even when the disclosure is negative.

Sample size	Sample size choice was based on previous studies (ref. Cao, Z., Li, D., Zhao, L. et al. https://doi.org/10.1038/s41467-022-29693-8; Ye, D., Shuhendler, A., Cui, L. et al. https://doi.org/10.1038/nchem.1920; Ai, X., Ho, C., Aw, J. et al. https://doi.org/10.1038/ncomms10432; Zhou, H., Guo, M., Li, j. et al. https://doi.org/10.1021/jacs.0c10245), not predetermined by a statistical method. Sample sizes were indicated in the legend of each Figure and Supplementary Figure.
Data exclusions	No data were excluded.
Replication	We confirm all attempts at replication were successful. Replicates were conducted for all experiments quantified as described in the Figure legends.
Randomization	All samples were randomly allocated into experimental groups.
Blinding	Investigators were not blinded for nanomaterial synthesis, because determination of nanoparticles concentrations are considered as objective measures, not subject to bias. For in vivo experiments, the investigators were blinded to group allocation during data collection and analysis.

Reporting for specific materials, systems and methods

We require information from authors about some types of materials, experimental systems and methods used in many studies. Here, indicate whether each material, system or method listed is relevant to your study. If you are not sure if a list item applies to your research, read the appropriate section before selecting a response.

Materials & experimental systems

n/a	Involved in the study	
x		Antibodies
	x	Eukaryotic cell lines
x		Palaeontology and archaeology
	x	Animals and other organisms
x		Clinical data
x		Dual use research of concern
x		Plants

Methods

n/a	Involved in the study	
x		ChIP-seq
x		Flow cytometry
x		MRI-based neuroimaging

Eukaryotic cell lines

Policy information about cell lines and Sex and Gender in Research

Cell line source(s)	MDA-MB-468 cells and 293T cells were obtained from Procell Life Science&Technology Co.,Ltd (CL-0290B, Wuhan, China).
Authentication	Cells were identified by Short Tandem Repeat (STR) method.
Mycoplasma contamination	The cell line was tested negative for mycoplasm contamination per suppliers.
Commonly misidentified lines (See ICLAC register)	No commonly misidentified lines were used.

Animals and other research organisms

Policy information about studies involving animals; ARRIVE guidelines recommended for reporting animal research, and Sex and Gender in Research

Laboratory animals	BALB/c-nude mice (female, 6-8 weeks) were purchased from Gempharmatech Co. Ltd (China). All the experimental animals were housed in specific pathogen-free conditions with a 12h light and 12h dark cycle, 25°C room temperature and 50.0±5.0% humidity and had access to food and water ad libitum.
Wild animals	Wild animals were not involved in this study.
Reporting on sex	Female mice were used in the studies of in vitro and in vivo experiments, and there was no other sex bias in the animals used.
Field-collected samples	Field-collected samples were not involved in this study.
Ethics oversight	The use and care of the mice were in accordance with the guidelines of the Institutional Animal Care and Use Committee of Shanghai Jiao Tong University. All procedures were approved by the Institutional Animal Care and Use Committee of Shanghai Jiao Tong University and Zhejiang University School of Medicine. Number is A2022006 approved experiments.

Note that full information on the approval of the study protocol must also be provided in the manuscript.

Plants

Seed stocks	N/A
Novel plant genotypes	N/A
Authentication	N/A
